# *Clostridium difficile* Colonizes Alternative Nutrient Niches during Infection across Distinct Murine Gut Microbiomes

**DOI:** 10.1128/mSystems.00063-17

**Published:** 2017-07-25

**Authors:** Matthew L. Jenior, Jhansi L. Leslie, Vincent B. Young, Patrick D. Schloss

**Affiliations:** a Department of Microbiology and Immunology, University of Michigan, Ann Arbor, Michigan, USA; b Department of Internal Medicine, Division of Infectious Diseases, University of Michigan, Ann Arbor, Michigan, USA; G. W. Hooper Research Foundation

**Keywords:** *Clostridium difficile*, infection, microbiome, metabolomics, network modeling, systems biology, transcriptomics

## Abstract

*Clostridium difficile* is the largest single cause of hospital-acquired infection in the United States. A major risk factor for *Clostridium difficile* infection (CDI) is prior exposure to antibiotics, as they disrupt the gut bacterial community which protects from *C. difficile* colonization. Multiple antibiotic classes have been associated with CDI susceptibility, many leading to distinct community structures stemming from variation in bacterial targets of action. These community structures present separate metabolic challenges to *C. difficile*. Therefore, we hypothesized that the pathogen adapts its physiology to the nutrients within different gut environments. Utilizing an *in vivo* CDI model, we demonstrated that *C. difficile* highly colonized ceca of mice pretreated with any of three antibiotics from distinct classes. Levels of *C. difficile* spore formation and toxin activity varied between animals based on the antibiotic pretreatment. These physiologic processes in *C. difficile* are partially regulated by environmental nutrient concentrations. To investigate metabolic responses of the bacterium *in vivo*, we performed transcriptomic analysis of *C. difficile* from ceca of infected mice across pretreatments. This revealed heterogeneous expression in numerous catabolic pathways for diverse growth substrates. To assess which resources *C. difficile* exploited, we developed a genome-scale metabolic model with a transcriptome-enabled metabolite scoring algorithm integrating network architecture. This platform identified nutrients that *C. difficile* used preferentially between pretreatments, which were validated through untargeted mass spectrometry of each microbiome. Our results supported the hypothesis that *C. difficile* inhabits alternative nutrient niches across cecal microbiomes with increased preference for nitrogen-containing carbon sources, particularly Stickland fermentation substrates and host-derived glycans.

**IMPORTANCE** Infection by the bacterium *Clostridium difficile* causes an inflammatory diarrheal disease which can become life threatening and has grown to be the most prevalent nosocomial infection. Susceptibility to *C. difficile* infection is strongly associated with previous antibiotic treatment, which disrupts the gut microbiota and reduces its ability to prevent colonization. In this study, we demonstrated that *C. difficile* altered pathogenesis between hosts pretreated with antibiotics from separate classes and exploited different nutrient sources across these environments. Our metabolite score calculation also provides a platform to study nutrient requirements of pathogens during an infection. Our results suggest that *C. difficile* colonization resistance is mediated by multiple groups of bacteria competing for several subsets of nutrients and could explain why total reintroduction of competitors through fecal microbial transplant currently is the most effective treatment for recurrent CDI. This work could ultimately contribute to the identification of targeted, context-dependent measures that prevent or reduce *C. difficile* colonization, including pre- and probiotic therapies.

## Abstract (precis)

Infection by the bacterium *Clostridium difficile* causes an inflammatory diarrheal disease which can become life threatening and has grown to be the most prevalent nosocomial infection. Susceptibility to *C. difficile* infection is strongly associated with previous antibiotic treatment, which disrupts the gut microbiota and reduces its ability to prevent colonization. In this study, we demonstrated that *C. difficile* altered pathogenesis between hosts pretreated with antibiotics from separate classes and exploited different nutrient sources across these environments. Our metabolite score calculation also provides a platform to study nutrient requirements of pathogens during an infection. Our results suggest that *C. difficile* colonization resistance is mediated by multiple groups of bacteria competing for several subsets of nutrients and could explain why total reintroduction of competitors through fecal microbial transplant currently is the most effective treatment for recurrent CDI. This work could ultimately contribute to the identification of targeted, context-dependent measures that prevent or reduce *C. difficile* colonization, including pre- and probiotic therapies.

## INTRODUCTION

Infection by the Gram-positive, spore-forming bacterium *Clostridium difficile* has increased in both prevalence and severity across numerous countries during the last decade (1). In the United States, *C. difficile* was estimated to have caused >500,000 infections and resulted in ~$4.8 billion worth of acute care costs in 2014 (2). *C. difficile* infection (CDI) causes an array of toxin-mediated symptoms ranging from abdominal pain and diarrhea to the more severe conditions pseudomembranous colitis and toxic megacolon. Prior treatment with antibiotics is the most common risk factor associated with development of CDI (3). Antibiotics contribute to an individual’s susceptibility to CDI by disrupting the gut microbiota (4). In mouse models, multiple antibiotics can induce susceptibility to *C. difficile* colonization (5–7). Notably, each antibiotic resulted in unique gut bacterial communities that permitted high levels of *C. difficile* colonization. Others have also shown that antibiotics from multiple classes also alter the gut metabolome, increasing the concentrations of some *C. difficile* growth substrates (6, 8–10). The ability of an unaltered murine gut community to exclude *C. difficile* colonization supports the nutrient niche hypothesis, which states that an organism must be able to utilize a subset of available resources better than all competitors to colonize the intestine (11, 12). Taken together, these results are a strong indication that the healthy gut microbiota inhibits the growth of *C. difficile* by limiting the availability of the substrates that it needs to grow.

Based on genomic and *in vitro* growth characteristics, *C. difficile* appears able to adapt to a variety of nutrient niches (13). *C. difficile* has a relatively large and mosaic genome, can utilize a variety of growth substrates, and possesses a diverse array of hosts (6, 14–16). These qualities are hallmarks of ecological generalists (17). *C. difficile* has also been shown to integrate signals from multiple forms of carbon metabolism to regulate its pathogenesis. *In vitro* transcriptomic analyses suggest that high concentrations of easily metabolized carbon sources, such as glucose or amino acids, inhibit toxin gene expression and sporulation (18, 19). Other studies have indicated that other aspects of *C. difficile* metabolism may be influenced through environmental nutrient concentration-sensitive global transcriptional regulators such as CodY and CcpA (20, 21). These analyses focused on *in vitro* growth (22, 23) or colonization of germfree (GF) mice (14, 21). Although these analyses are informative, they either are directed toward the expression of pathogenicity factors or lack the context of the gut microbiota against which *C. difficile* must compete for substrates. Metabolomic investigations have also been used to assay changes in bacterial metabolism as they relate to CDI and have characterized the levels of germinants and growth substrates (6, 10); however, metabolomic approaches are unable to attribute a metabolite to specific organisms in the gut community. Thus, metabolomics more closely represents the echoes of total community metabolism, not the currently active processes of any one population. It has thus far not been possible to study the metabolism of *C. difficile in vivo*. To overcome these limitations, we implemented transcriptomic and untargeted metabolomic analyses of *C. difficile* and the surrounding environment to better understand the active metabolic pathways in a model of infection. Based on the ability of *C. difficile* to grow on a diverse array of carbon sources and its ability to colonize a variety of communities, we hypothesized that *C. difficile* adapts its metabolism to fit the context of the environment that it is attempting to colonize. To test this hypothesis, we employed a mouse model of infection to compare the metabolic responses of *C. difficile* to the gut environment caused by three antibiotics from distinct classes. By characterizing a transcriptome-enabled metabolic model of *C. difficile* and changes in the metabolome of each respective environment, we were able to generate a systems model to directly test the nutrient niche hypothesis.

## RESULTS

### Levels of *C. difficile* sporulation and toxin activity vary among different microbiomes.

**FIG 1:**
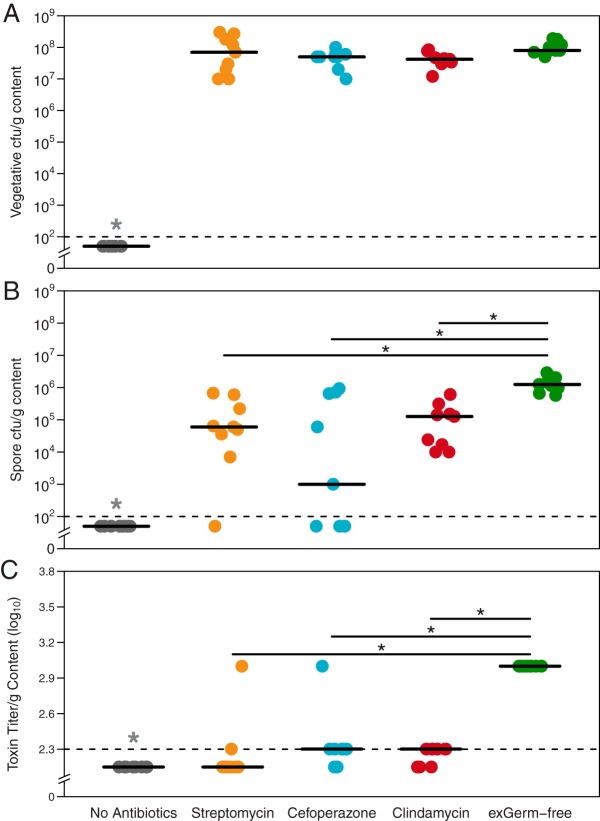
Gut environment context affects *C. difficile* sporulation and toxin activity. Quantification of spore CFU and toxin titer from cecal content of infected mice (*n* = 9 per group). (A) Vegetative *C. difficile* CFU per gram of cecal content (*P* > 0.05). (B) *C. difficile* spore CFU per gram of cecal content. (C) Toxin titer from cecal content measured by activity in Vero cell rounding assay. Dotted lines denote limits of detection (LOD). Values for undetectable points were imputed as half the LOD for calculation of significant differences. Significance (*P* < 0.05), denoted by a single asterisk, was determined with the Wilcoxon signed-rank test with the Benjamini-Hochberg correction.

Conventionally reared specific-pathogen-free (SPF) mice were treated with either streptomycin, cefoperazone, or clindamycin (Table 1; also see Fig. S1 in the supplemental material). These antibiotics were selected because they each have distinct and significant impacts on the structure of the cecal microbiome (Fig. S2A and B). We challenged the antibiotic-treated mice and germfree (exGF) mice with *C. difficile* strain 630 to understand the pathogen’s physiology with and without other microbiota. This toxigenic strain of *C. difficile* was chosen for its moderate clinical severity in mouse models (24) and well-annotated genome (25). After infection, we measured sporulation and toxin production at 18 h postinoculation. That time point corresponded with when another laboratory strain of *C. difficile* reached its maximum vegetative cell density in the cecum with limited sporulation (26). There was not a significant difference in the numbers of vegetative *C. difficile* cells in the ceca of mice pretreated with any of the three antibiotics (Fig. 1A). All antibiotic-treated and exGF mice were colonized to ~1 × 10^8^ CFU per gram of cecal content, while untreated mice maintained colonization resistance to *C. difficile* (Fig. 1A). Despite having the same number of vegetative *C. difficile* cells, more spores were detected in exGF mice than in the antibiotic-pretreated mice (*P* = 0.003, 0.004, and 0.003) (Fig. 1B). Toxin activity was relatively low across each group tested compared to previous studies (24, 27). The low activity was likely the result of the early sampling time point during infection. In spite of this, the toxin activity in exGF animals was significantly higher than that in any other colonized group (all *P* values were <0.001), with slight variation between antibiotic pretreatment groups (Fig. 1C). These results showed that *C. difficile* colonized different communities to consistently high levels but had subtle variation in sporulation and toxin activity between distinct antibiotic-pretreated environments. As activation of both traits has been linked to recognition of distinct nutrient source concentrations in the environment (28, 29), we hypothesized that *C. difficile* was utilizing different growth substrates across the conditions tested. To investigate the physiology of *C. difficile* when colonizing distinct susceptible gut environments, we performed whole-transcriptome analysis of *C. difficile* from the cecal content of the same mice used to measure *C. difficile* density and toxin activity.

10.1128/mSystems.00063-17.1 FIG S1  Experimental timelines for mouse model pretreatments and *C. difficile* infection. Nine wild-type C57BL/6 mice across 3 cages were included in each treatment group. (A and B) Streptomycin (A) or cefoperazone (B) administered *ad libitum* in drinking water for 5 days with 2-day recovery with untreated drinking water before infection. (C) A single clindamycin intraperitoneal injection 1 day prior to infection. (D) No antibiotic pretreatment (for both SPF control and GF mice). If no antibiotics were administered in the drinking water, mice were given untreated drinking water for the duration of the experiment beginning 7 days prior to infection. At the time of infection, mice were challenged with 1 × 10^3^ spores of *C*. *difficile* strain 630. Euthanization and necropsy were done 18 h postchallenge, and cecal content was then collected. Download FIG S1, PDF file, 0.01 MB. Copyright © 2017 Jenior et al. 2017 Jenior et al. This content is distributed under the terms of the Creative Commons Attribution 4.0 International license.

10.1128/mSystems.00063-17.2 FIG S2  Analysis of bacterial community structure resulting from antibiotic treatment. Results from 16S rRNA gene amplicon sequencing from bacterial communities of cecal content in both mock-infected and *C. difficile* 630-infected animals 18 h postinfection across pretreatment models. (A) Nonmetric multidimensional scaling (NMDS) ordination based on theta_YC_ distances for the gut microbiome of all SPF mice used in these experiments (*n* = 36). All treatment groups are significantly different from each other by AMOVA (*P* < 0.001). (B) Inverse Simpson diversity for each cecal community from the mice in panel A. Cecal communities from mice not treated with any antibiotics are significantly more diverse than those from mice under any antibiotic-pretreated condition (*P* < 0.001). (C) Representation of 16S amplicon reads contributed by *C. difficile* under each sequenced condition compared to the total bacterial community. The percentage listed at the top of each group is the proportion of the total community represented by *C. difficile*. Significantly lower values for *C. difficile* were detected under each condition (*P* < 0.001). Download FIG S2, PDF file, 0.01 MB. Copyright © 2017 Jenior et al. 2017 Jenior et al. This content is distributed under the terms of the Creative Commons Attribution 4.0 International license.

**TABLE 1:** Antibiotics used in *C. difficile* murine infection models

Antibiotic	Class	Target	Activity	Administration	Dosage
Cefoperazone	Cephalosporin (third generation)	Primarily Gram-positive bacteria, with increased activity against Gram-negative bacteria	Irreversible cross-linking of bacterial transpeptidases to peptidoglycan and prevention of cell wall synthesis	Drinking water *ad libitum* for 5 days and 2 days of untreated drinking water prior to infection	0.5 mg/ml drinking water
Streptomycin	Aminoglycoside	Active against most Gram-negative aerobic and facultative anaerobic bacilli	Protein synthesis inhibition through binding the 30S portion of the 70S ribosomal subunit	Drinking water *ad libitum* for 5 days and 2 days of untreated drinking water prior to infection	5.0 mg/ml drinking water
Clindamycin	Lincosamide	Primarily active against Gram-positive bacteria, most anaerobic bacteria, and some mycoplasmas	Protein synthesis inhibition through binding to the 23S portion of the 50S ribosomal subunit	Intraperitoneal injection 24 h prior to infection	10 mg/kg body weight

### *C. difficile* alters its gene expression pathways when colonizing distinct antibiotic-pretreated environments.

Utilizing aliquots of cecal content from the same mice that were in the previous assays, we measured differential expression of specific genes associated with *in vivo* phenotype changes reported in previous studies with a transcriptome-sequencing (RNA-Seq)-based approach. Microarray-based gene expression measurement was not a viable alternative to sequencing as the amount of background orthologous transcription from other bacterial species would contribute to nonspecific binding and bias the true *C. difficile* signal. Therefore, we employed RNA-Seq to quantify *C. difficile*-specific transcription. *C. difficile* represented a small percentage of the community in each colonized environment (Fig. S2C), which made it impossible to sequence the transcriptome of individual mice due to the depth required to sufficiently sample the transcripts of *C. difficile*. This required the generation of a single transcriptome per condition using pooled mRNA from all mice within each pretreatment group. Following sequencing, read curation, and stringent mapping to *C. difficile* strain 630 genes (see Materials and Methods), we implemented two steps of abundance normalization to compare expression between groups. Transcript abundances for each target gene were first corrected to both read length and target gene length, which resulted in an average per-base expression level for each. Adjusted values were then down-sampled to the same total read abundance for each mapping effort, allowing for even comparison between the conditions. Additionally, before proceeding with the analysis we assessed variation in expression of select bacterial housekeeping genes across treatment groups (Fig. S3A). Due to the heterogeneity of *C. difficile* reference genes across strains (30), we chose DNA gyrase subunit A (GyrA), threonyl-tRNA synthetase (ThrS), and ATP-dependent Clp protease (ClpP) because they are conserved across bacterial phyla and have been commonly utilized as standards for numerous transcriptional studies (14, 31, 32). We observed consistent expression for each of the housekeeping genes across treatments, which indicated that our results were more likely to be a true reflection of *C. difficile* expression *in vivo*. We then focused on select genes previously demonstrated to have altered transcription based on environmental cues, including several key sigma factors (29) and downstream genes involved in sporulation (33), toxin production (34), and quorum sensing (35) (Fig. S4). Comparing these data to results described above, toxin gene expression seemed to vary between conditions more than the activity data would suggest (Fig. S4B). However, the abundance of cDNA transcript recruited within this mapping effort relative to the toxin genes was very low, which would agree with the generally low levels of toxin activity detectable across treatment groups (Fig. 1C). For the other gene categories, consistent trends across pretreatments were not apparent through this analysis, and so we decided to shift our focus toward differences in metabolic pathways that were more explicitly involved in the breakdown of environmentally acquired nutrients.

10.1128/mSystems.00063-17.3 FIG S3  Levels of within-group variation across data sets generated for this study. (A) Normalized transcript abundance of select housekeeping and central metabolism genes. I, housekeeping genes; DNA gyrase subunit A (GyrA), threonyl-tRNA synthetase (ThrS), and ATP-dependent Clp protease (ClpP). II, genes in separate metabolic pathways that contribute to input substrate score; enolase, glycine reductase (GrdA), and d-proline reductase (PrdA). (B) Median sample variance for vegetative *C. difficile* CFU from each colonized condition. (C) Median and interquartile range of the sample variance of OTU abundances from 16S rRNA gene sequencing; sample variances for each OTU were calculated individually prior to summary statistic calculations. (D) Median and interquartile range of the sample variance of scaled intensities from untargeted metabolomic analysis; sample variances for each metabolite were in the same fashion as those with OTU abundances. Data (other than transcriptomic results) were collected from the same nine animals per group (*n* = 9). Download FIG S3, PDF file, 0.01 MB. Copyright © 2017 Jenior et al. 2017 Jenior et al. This content is distributed under the terms of the Creative Commons Attribution 4.0 International license.

10.1128/mSystems.00063-17.4 FIG S4  Select *C. difficile* gene set expression levels compared between treatment groups. Relative abundances of *C. difficile* transcript for specific genes of interest. (A) Transcription for select genes from the *C. difficile* sporulation pathway with the greatest variation in expression between the conditions tested. (B) Relative abundances of transcript for genes that encode effector proteins from the *C. difficile* pathogenicity locus. (C) Transcript abundances for genes associated with quorum sensing in *C. difficile*. (D) Transcript relative abundance of select sigma factors whose expression or activity is influenced by environmental metabolite concentrations. Asterisks indicate genes from which transcript was undetectable. Download FIG S4, PDF file, 0.01 MB. Copyright © 2017 Jenior et al. 2017 Jenior et al. This content is distributed under the terms of the Creative Commons Attribution 4.0 International license.

**FIG 2:**
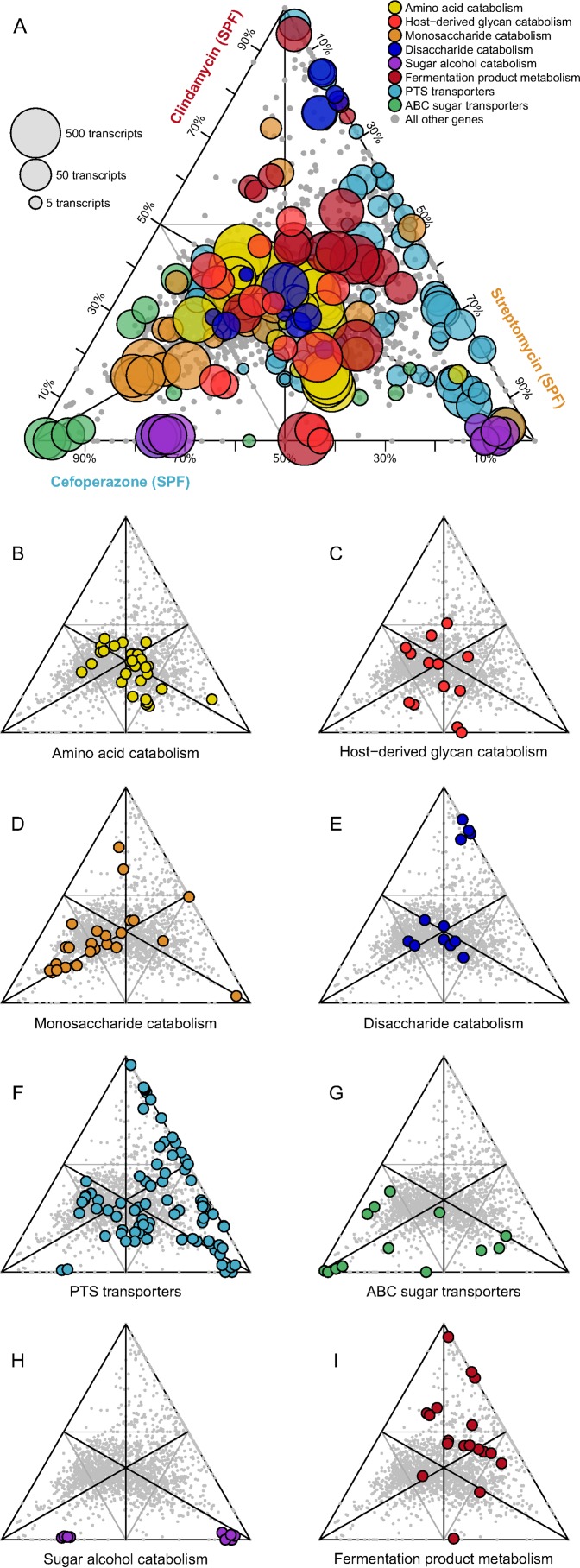
*C. difficile* alters expression metabolic pathways between antibiotic pretreatment models. Each point in the ternary plot represents a unique gene from the annotated genome of *C. difficile* strain 630. The position reflects the ratio of median rarefied transcript abundance for that gene between the three colonized antibiotic pretreatment models. Genes from specific metabolic pathways of interest are labeled, and transcription from all other genes is shown in gray. (A) The size of highlighted points is relative to the highest transcript abundance among the antibiotic pretreatments for each gene. (B to I) Categories of metabolism are displayed separately. Genes, annotations, and normalized transcript abundances can be found in Table S1.

We chose to assess transcriptional differences in several specific families of genes known to contribute to different aspects of *C. difficile* metabolism (Fig. 2A; Table S1). Genes involved in amino acid catabolism, including those that encoded enzymes involved in Stickland fermentation and general peptidases, had the highest level of expression across all pretreatments. Stickland fermentation refers to the coupled fermentation of amino acid pairs in which one is deaminated and the other is reduced to ultimately generate ATP (36). This suggested that *C. difficile* catabolized environmental amino acids during infection, regardless of the structure of the surrounding community. Although there were gene categories that were equally expressed across conditions in spite of the community differences, there were patterns of expression for certain gene families and specific genes that were specific to each antibiotic pretreatment. In mice pretreated with cefoperazone, *C. difficile* tended to have higher expression of genes in the ABC sugar transporter and sugar alcohol catabolism (e.g., mannitol) families and lower expression of genes in the phosphotransferase system (PTS) transporter family than the other pretreatment groups. In mice pretreated with clindamycin, *C. difficile* tended to have higher expression of genes from disaccharide catabolism (e.g., beta-galactosidases and trehalose/maltose/cellibiose hydrolases), fermentation product metabolism (including consumption or production of acetate, lactate, butyrate, succinate, ethanol, and butanol), and PTS transporter families. Genes from the sugar alcohol catabolism and ABC sugar transporter families were not highly expressed in the clindamycin-pretreated mice. Finally, in mice pretreated with streptomycin, *C. difficile* had higher levels of expression of genes from the sugar alcohol catabolism (e.g., sorbitol) and PTS transporter families. Combined, these results suggested that while catabolism of amino acids and specific carbohydrates is a core component of the *C. difficile* nutritional strategy during infection, *C. difficile* adapted its metabolism across different susceptible environments.

10.1128/mSystems.00063-17.6 TABLE S1  Specific genes and normalized cDNA read abundances included in the analysis reported in Fig. 2. Transcript abundances reported in each of the antibiotic-associated columns were first normalized to both sequencing read length and target gene length. Each of the three groups was then evenly subsampled to an equal total sequence abundance of 13,000 reads to allow for comparability between groups. Additional columns indicate specific gene annotation (gene, pathways, and KEDD_ID) as well as which group each gene belongs to for ternary plot (family). Download TABLE S1, XLSX file, 0.02 MB. Copyright © 2017 Jenior et al. 2017 Jenior et al. This content is distributed under the terms of the Creative Commons Attribution 4.0 International license.

### Genome-scale metabolic model structure underscores known *C. difficile* physiology.

**FIG 3:**
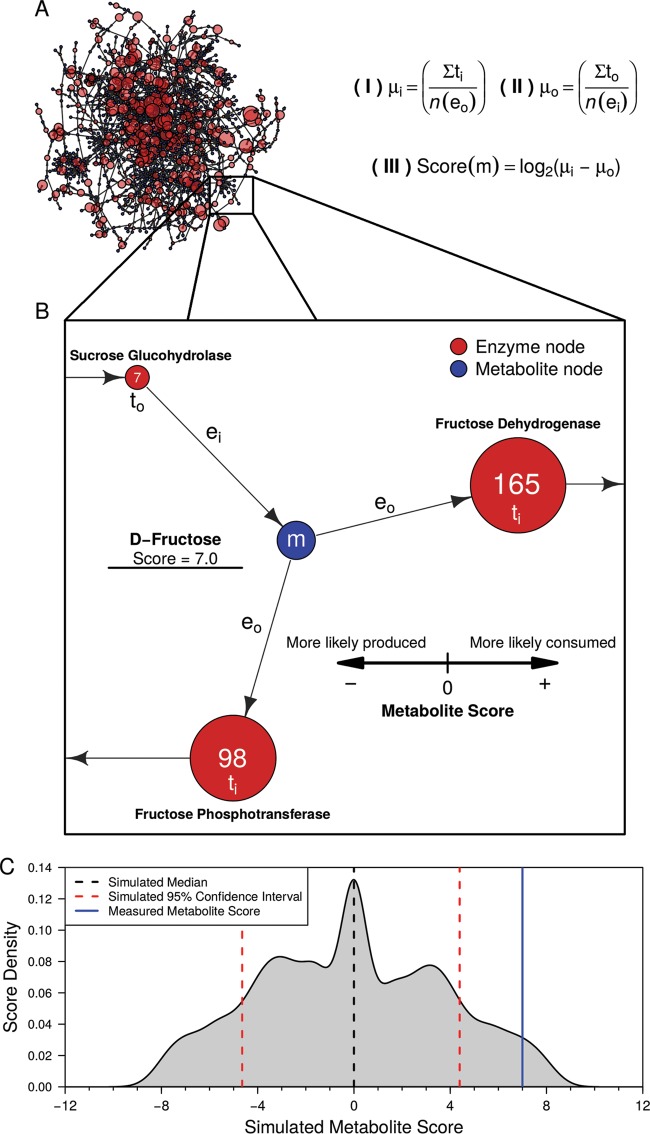
*C. difficile* strain 630 genome-enabled bipartite metabolic network architecture and transcriptomic-enabled metabolite score calculation. (A) Largest component from the bipartite genome-enabled model of *C. difficile* strain 630. Enzyme node sizes reflect the levels of detectable transcript from each gene. Metabolite score algorithm components: I, average transcription of reactions consuming a metabolite; II, average transcription of reactions producing a metabolite; III, difference of consumption and production. (B) The expanded window displays a partial example of d-fructose score calculation. Values in the red nodes represent normalized transcript reads mapping to enzymes. (C) Example of 10,000-fold Monte Carlo simulation results corresponding to a significant metabolite score for *m*.

Because multiple enzymes can utilize the same input substrates within a single organism, we decided to implement a metabolic network-based approach to further investigate which metabolites were differentially utilized between conditions by *C. difficile*. This approach is more robust at identifying reporter metabolites than assessing individual gene transcription because if the amount of a single enzyme that acts on a substrate decreases and yet the amounts of others that also act on that substrate increase, those changes are more readily apparent in the context of a network. To perform this analysis, we created a generalizable tool to generate *de novo* genome-enabled bipartite metabolic models with directed enzymatic reactions of bacterial species using KEGG gene and biochemical reaction annotations. We implemented this platform using the genome of *C. difficile* strain 630 (Fig. 3A), with enzymes and metabolites represented by nodes and their interactions represented by directed connecting edges. The *C. difficile* strain 630 network contained a total of 447 enzymes and 758 metabolites, with 2,135 directed edges (Fig. 3A). To validate our metabolic network, we analyzed network topology by calculating two metrics of centrality, betweenness centrality (BC) and closeness centrality (CC), to determine which nodes are critical to the structure of the metabolic network and if these patterns reflect known biology (Table S3). Both metrics utilize shortest paths, which refer to the lowest possible number of network connections that lie between two given nodes. The BC of each node is the fraction of shortest paths that pass through that node and connect all other potential pairs of nodes. In biological terms, this refers to the amount of influence that a given hub has on the overall flow of metabolism (37). Similarly, CC is the reciprocal sum of the lengths of shortest paths included in each node’s BC. This value demonstrates how essential a given node is to the overall structure of the metabolic network (38). Metabolic network structural studies of *Escherichia coli* have found that metabolites with the highest centrality calculations are involved in fundamental processes in metabolism, namely, glycolysis and the citrate acid cycle pathway (39). As such, these metrics allow for assessment of the degree to which a metabolic network accurately depicts established principles of bacterial metabolism.

10.1128/mSystems.00063-17.7 TABLE S2  Normalized cDNA read abundances, gene annotations, and enzymatic reaction information used for metabolic model building for *C. difficile* strain 630 KEGG orthologs across colonized conditions. All KEGG orthologs included in the *C. difficile* strain 630 KEGG genome annotation (2015) were included in this analysis. Read abundances were normalized as previously outlined to sequencing read length, target gene length, and even total sampling between groups. Also included are individual enzyme annotations for each KEGG ortholog, as well as the associated biochemical reaction information extracted from reaction/reaction_mapformula.lst from KEGG. Together, KEGG ortholog and enzymatic reaction data were used to reconstruct the metabolic network of *C. difficile* strain 630 in presented analyses. Download TABLE S2, XLSX file, 0.1 MB. Copyright © 2017 Jenior et al. 2017 Jenior et al. This content is distributed under the terms of the Creative Commons Attribution 4.0 International license.

10.1128/mSystems.00063-17.8 TABLE S3  Topology metrics for enzyme and metabolite nodes in the *C. difficile* strain 630 metabolic network. Topology analysis of the metabolic network assembled for this study was performed in the absence of transcriptomic data to assess the quality of the *de novo*-assembled network in its reflection of known bacterial metabolism patterns. Enzyme and metabolite node analyses are presented on separate tabs. Centrality metrics and brief explanations are as follows: degree is the total number of connections for a given node (both incoming and outgoing), betweenness is the number of shortest paths connecting all other node pairs that pass through the node of interest, and closeness is the inverse sum of shortest path lengths that pass through the node of interest. Combined, these calculations inform how strongly connected a node is and how vital it is to overall network structure. Download TABLE S3, XLSX file, 0.1 MB. Copyright © 2017 Jenior et al. 2017 Jenior et al. This content is distributed under the terms of the Creative Commons Attribution 4.0 International license.

Following application of both methods, we found 5 enzymes that were shared between the top 10 enzymes from BC and CC calculations (2-dehydro-3-deoxyphosphogluconate aldolase, aspartate aminotransferase, pyruvate-flavodoxin oxidoreductase, formate C-acetyltransferase, and 1-deoxy-d-xylulose-5-phosphate synthase). These enzymes primarily participate in core processes, including glycolysis, the pentose phosphate pathway, or the citric acid cycle. Upon analysis of the other 15 high-scoring enzymes combined from BC and CC analyses, the majority were also components of the abovementioned pathways, as well as several for the metabolism of amino acids (Table S3). Similarly, the intersection of those substrates with both high BC and high CC values indicated that 6 metabolites were central to the metabolism of *C. difficile* (pyruvate, acetyl coenzyme A [CoA], 2-oxoglutarate, d-4-hydroxy-2-oxoglutarate, d-glyceraldehyde 3-phosphate, and l-glutamate). Not only are these members of glycolysis and the citric acid cycle, but pyruvate, acetyl-CoA, and l-glutamate contribute to numerous intracellular pathways as forms of biological “currency” (39). Notably absent from the most well-connected metabolites were molecules like ATP or NADH. Their exclusion is likely a by-product of the KEGG LIGAND reference used for network construction, which excludes cofactors from most biochemical reactions. While this may be a limitation of certain analyses, our study was not affected as the primary interest was in those substrates acquired from the environment. These results reflected the defined biological patterns of *C. difficile* and were therefore a viable platform to study metabolism of the pathogen.

### Metabolite score algorithm reveals adaptive nutritional strategies of *C. difficile* during infection of distinct environments.

We next sought to incorporate the transcriptomic results into the metabolic model to infer which metabolites *C. difficile* most likely utilized from a given environment. To accomplish this, we mapped normalized transcript abundances to the enzyme nodes in the network. Similar approaches have been previously successful in demonstrating that transcript abundance data can be utilized through the lens of genome-scale metabolic networks to accurately predict microbial metabolic responses to environmental perturbation and identify reporter metabolites of changes (40). In our system, the score of each metabolite was measured as the log_2_-transformed difference in average transcript levels of enzymes that use the metabolite as a substrate and those that generate the metabolite as a product (Fig. 3B). A metabolite with a high score was likely obtained from the environment because the expression of genes for enzymes that produce the metabolite was low. It is important to note that molecules that were more likely produced in our model were not necessarily likely to be released to the environment. Our models do not include the synthesis of large macromolecules (i.e., long polypeptides or cytoskeleton) and should therefore be utilized to consider only metabolites that were inputs to a network. Due to the previously mentioned limited technical replication of sequencing efforts, we adopted a Monte Carlo-style simulation for iterative random transcriptome comparison to provide statistical validation of our network-based findings. This process generated random score distributions for each metabolite node in the network, which made it possible to calculate a confidence interval that represented random noise for each metabolite. This ultimately allowed for assessment of the probability that a given metabolite was excluded from the associated null distribution (Fig. 3C).

**FIG 4:**
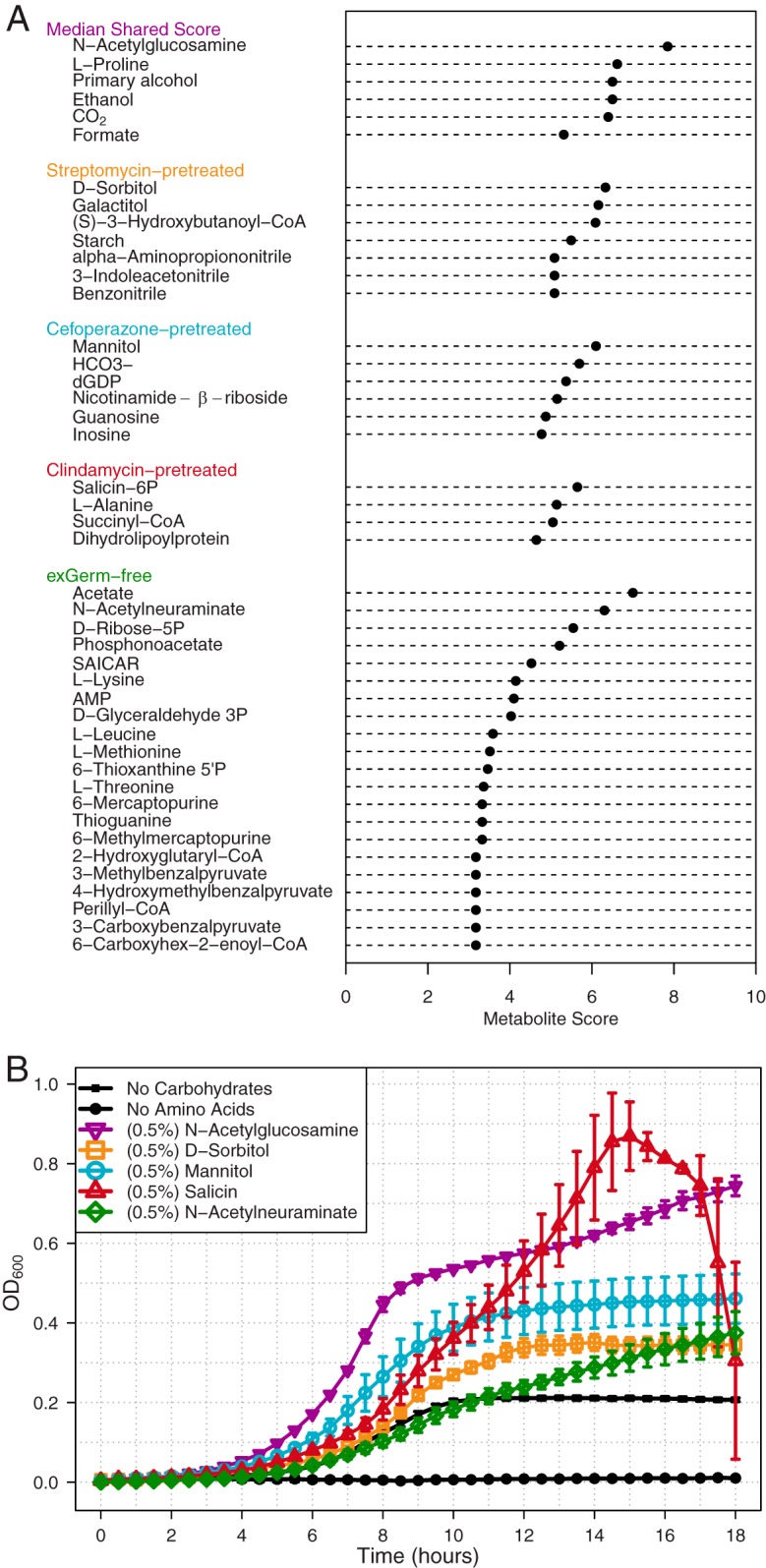
Metabolic network analysis reveals differential carbon source utilization by *C. difficile* across infections. Reported metabolite scores were calculated to have <2.5% probability of being included in the associated random score distribution. Analysis was performed using the 40 highest-scoring metabolites from each condition. (A) The shared metabolite score represents the median score of metabolites that consistently scored highly among all infected conditions. Below the conserved patterns are shown the distinct metabolites for each group’s subset. (B) Eighteen hours of *C. difficile* strain 630 *in vitro* growth validating substrates from network analysis. All statistical comparison was performed relative to the no-carbohydrate control (all *P* values were <0.001). Significance was determined with one-way ANOVA with the Benjamini-Hochberg correction.

To identify the metabolites that were most essential for *C. difficile* growth, regardless of the environment, we cross-referenced the 40 highest-scoring metabolites from each treatment group (Fig. 4A). *N*-Acetylglucosamine (GlcNAc) was found to have the highest median score of all shared metabolites and has been shown to be a readily available source of carbon and nitrogen, which can be limiting in the gut (21). We went on to confirm that our strain of *C. difficile* could metabolize GlcNAc for growth (Fig. 4B; Table S5) in *C. difficile* minimal medium (41). The Stickland fermentation acceptor proline scored highly across all conditions (42). *C. difficile* is auxotrophic for not only proline but also cysteine, leucine, isoleucine, tryptophan, and valine, which prevented testing for *in vitro* growth changes on proline despite providing for modest growth in the no-carbohydrate control. Previous analysis of *C. difficile* colonizing GF mice under monoassociated conditions indicated that *C. difficile* uses both sets of metabolites (21); however, use of these metabolites in the context of a complex community of potential competitors has not been observed. This analysis indicated that these metabolites might be an integral component of the nutrient niche for *C. difficile*.

10.1128/mSystems.00063-17.9 TABLE S4  Metabolites with significant metabolite scores for *C. difficile* under each colonized condition. Each tab represents those metabolites found to exceed the significance cutoffs for *C. difficile* strain 630 after colonization of each of the respective susceptible states. These thresholds were set for each metabolite independently through Monte Carlo simulation as outlined in Fig. 3C. A *P* value of <0.05 corresponded to a metabolite scoring outside the 95% confidence interval in the random distribution, and a *P* value of <0.01 corresponded to those outside the 99% confidence interval. Confidence interval calculations for nonnormal distributions were performed as defined in reference 59. Download TABLE S4, XLSX file, 0.1 MB. Copyright © 2017 Jenior et al. 2017 Jenior et al. This content is distributed under the terms of the Creative Commons Attribution 4.0 International license.

10.1128/mSystems.00063-17.10 TABLE S5  *In vitro* growth analysis for *C. difficile* 630 with carbon sources identified by metabolic network algorithm. Analysis of growth on high-scoring carbon sources to identify possible differences in utilization efficiency. Download TABLE S5, XLSX file, 0.01 MB. Copyright © 2017 Jenior et al. 2017 Jenior et al. This content is distributed under the terms of the Creative Commons Attribution 4.0 International license.

### *In vivo* metabolomic analysis supports that *C. difficile* consumes metabolites indicated by metabolic modeling.

To further validate the results of our metabolic model, we tested the effect of *C. difficile* on the metabolite pool in cecal contents from each antibiotic-pretreated and exGF mouse used in the previous analyses. This afforded us the ability to compare replicates within each treatment group. To measure metabolite concentrations, we utilized nontargeted ultraperformance liquid chromatography and mass spectrometry (UPLC-MS) to measure the relative *in vivo* concentrations of metabolites for each mouse under the conditions investigated, with special attention to those highlighted by high metabolite scores. We tested whether the susceptible communities had significantly different concentrations of each metabolite relative to untreated SPF mice and whether the presence of *C. difficile* affected the metabolite composition.

**FIG 5:**
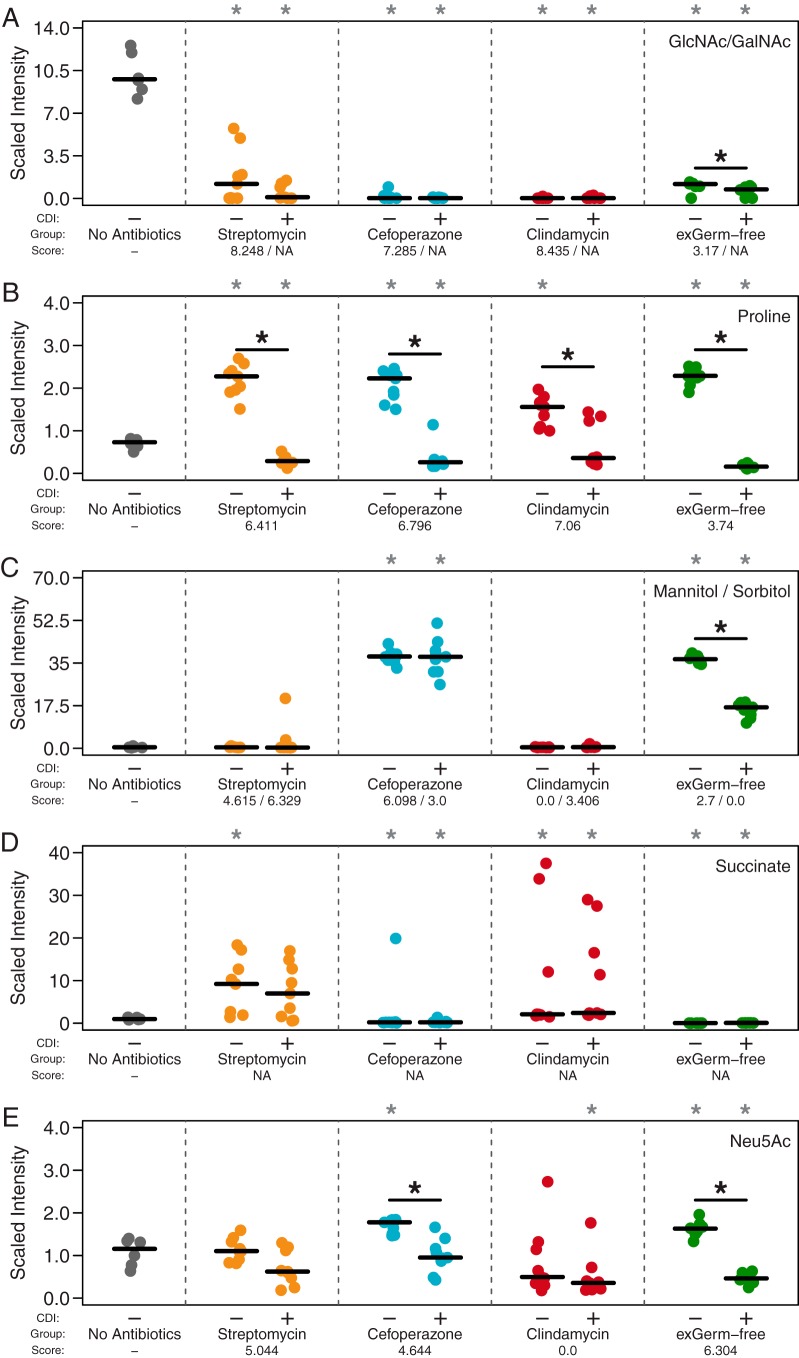
*In vivo* untargeted metabolomics support network-based metabolite scores and suggest nutrient preference hierarchy. Paired metabolites were quantified simultaneously as they differ only by chirality, making differentiation impossible. CDI status and *C. difficile* metabolite scores during infection are indicated below each panel. “NA” denotes metabolites that were not included in our metabolic model of *C. difficile* strain 630. Black asterisks inside the panels represent significant differences between mock- and *C. difficile*-infected groups within separate treatment groups (all *P* values were <0.05). Gray asterisks above each panel indicate significant differences from untreated SPF mice (all *P* values were <0.05). Significance was determined with the Wilcoxon signed-rank test with the Benjamini-Hochberg correction.

First, we compared the relative concentrations of highly scored metabolites in untreated SPF mice and antibiotic-pretreated mice in the absence of *C. difficile* (Fig. 5). We found that the relative concentration of GlcNAc was actually significantly lower under all susceptible conditions (Fig. 5A; all *P* values are <0.001). The concentrations of the Stickland fermentation acceptors proline (all *P* values are <0.05) and hydroxyproline (all *P* values are <0.05) were significantly higher in all susceptible environments tested (Fig. 5B and S5D). Succinyl-CoA, which is the direct precursor to succinate by succinyl-CoA transferases, scored highest in the clindamycin pretreatment (43). Succinate has been shown to support *C. difficile* growth *in vivo* through a synergistic relationship that requires at least one other bacterial species (9). As succinyl-CoA was not measured in our metabolomic assay, we instead found that succinate was indeed significantly higher in clindamycin-pretreated mice (Fig. 5D; all *P* values are <0.05). Among the cefoperazone-pretreated SPF and GF mice, we also found that the concentrations of mannitol/sorbitol (Fig. 5C), *N*-acetylneuraminate (Fig. 5E), and glycine (Fig. S5E) were significantly higher in cefoperazone-treated SPF and GF mice (all *P* values were <0.05). These results supported the assertion that susceptible mice had multiple nutrient niches that *C. difficile* was able to exploit.

10.1128/mSystems.00063-17.5 FIG S5  Change in *in vivo* concentrations of additional Stickland fermentation substrates. Comparison of concentrations for other Stickland fermentation substrates from *C. difficile*-infected and mock-infected mouse cecal content 18 h postinfection. Labels in the top left corner of each panel indicate whether the amino acid is a Stickland donor or acceptor. Black asterisks inside the panels denote significant differences between mock- and *C. difficile*-infected groups within separate treatment groups (all *P* values were <0.05). Gray asterisks above each panel indicate significant differences from untreated SPF mice (all *P* values were < 0.05). Download FIG S5, PDF file, 0.01 MB. Copyright © 2017 Jenior et al. 2017 Jenior et al. This content is distributed under the terms of the Creative Commons Attribution 4.0 International license.

Second, we compared relative concentrations of high-scoring metabolites during CDI and mock infection within each pretreatment group (Fig. 5). Both groups of host-derived glycans, GlcNAc/GalNAc (Fig. 5A) and Neu5Ac (Fig. 5E), were significantly lower when in the presence of *C. difficile* in exGF mice (*P* < 0.05 and 0.01). In agreement with the previous results, we found that the Stickland acceptors proline (Fig. 5B) and hydroxyproline (Fig. S5D) were significantly lower in every *C. difficile*-colonized environment (all *P* values were <0.05). Glycine, another preferred Stickland acceptor, was lower under each condition following infection, with a significant change in cefoperazone-pretreated mice (Fig. S5D; *P* < 0.05). The Stickland donors leucine and isoleucine were significantly lower under all infected conditions except in streptomycin-pretreated mice (Fig. S5; all *P* values were <0.05). These results supported the hypothesis that amino acids are an important energy source for *C. difficile* during infection. A significant difference was seen for mannitol/sorbitol in exGF mice (*P* < 0.01) but not in cefoperazone-pretreated mice (Fig. 5C). Although a lower concentration of succinate in both streptomycin- and clindamycin-pretreated mice was observed, neither was found to be significant. Overall, this metabolomic analysis supported our transcriptome-based metabolite score algorithm for predicting the metabolites utilized by *C. difficile* under different infection conditions. Results from metabolic modeling combined with untargeted metabolomic analysis also suggested a possible hierarchy of preferred growth substrates.

## DISCUSSION

The results presented here expand upon the previous understanding of *C. difficile* metabolism during infection by showing that the pathogen adapts its metabolism not only to life inside a host (14, 21) but also to the context of the specific gut environment in which it finds itself. Previous transcriptomic efforts to measure the response of *C. difficile* have characterized *in vivo* changes in metabolism following colonization of GF mice. In this study, we utilized a conventionally reared mouse model of infection to compare the responses of *C. difficile* to colonization in the context of varied gut communities generated by pretreatment with representatives from distinct classes of antibiotics. With these models, we identified subtle differences in sporulation and toxin activity between each antibiotic-pretreated condition. Transcriptomic sequencing of *C. difficile* across colonized environments indicated complex expression patterns of genes in catabolic pathways for a variety of carbon sources. Integration of transcriptomic data with genome-scale metabolic modeling allowed us to observe that *C. difficile* likely generated energy by metabolizing specific alternative carbon and nitrogen sources across colonized conditions. We also found that Stickland fermentation substrates and products, as well as the host-derived glycan *N*-acetylglucosamine, were consistently among the highest-scoring shared metabolites, which indicated that these metabolites were central to the *in vivo* nutritional strategy of *C. difficile*. To confirm our modeling-based results, we employed untargeted mass spectrometry that demonstrated greater availability of many metabolites highlighted by our algorithm in susceptible gut environments. Metabolomic analysis further revealed differential reduction of highly scored metabolites during CDI, which suggested a hierarchy for the utilization of certain growth nutrients.

Our interpretation of the positive trends that we observed between metabolite score and substrate availability across conditions was that the distinct antibiotic treatments eliminate alternative patterns of competitors for those nutrients in the gut of susceptible animals. These groups of bacteria are likely to be more specialized than *C. difficile* at acquiring those resources, supporting the nutrient niche hypothesis as a mechanism to explain the exclusion of *C. difficile* by the intact microbiota. By pursuing a more generalist behavior in terms of growth nutrient preferences, *C. difficile* has increased fitness for exploiting differentially perturbed gut communities. ExGF mice provided a partially controlled system of resource competition. Under this condition, Neu5Ac was found to be the highest-scoring substrate, and its concentrations were significantly higher during mock infection than following *C. difficile* colonization. A similar trend was also seen in cefoperazone-pretreated mice, implying that this antibiotic may have reduced the population density of the particular competitors for this niche. These data suggest that *C. difficile* may be less competitive for this host-derived glycan and have access only when certain competitors have had their densities reduced. In agreement with earlier research, we found that *C. difficile* likely fermented amino acids for energy during infection of GF mice in addition to host-derived glycan catabolism. Our results go on to support the idea that this metabolic strategy was conserved across all infection conditions tested. Several Stickland substrates that had consistently high metabolite scores, including alanine, leucine, and proline, indeed decreased in concentration during infection (Fig. 5B; see also Table S4 and Fig. S5A and B in the supplemental material). Fermentation of amino acids provides not only carbon and energy but also a source of nitrogen, which is a limited resource in the mammalian lower gastrointestinal tract (44). This makes Stickland fermentation a valuable metabolic strategy, and it stands to reason that *C. difficile* would use this strategy across all environments that it colonizes. This same principle may also extend to glycans harvested from the host mucus layer (GlcNAc and Neu5Ac), as they are another source of carbon and nitrogen which, despite augmented release by members of the microbiota, would be present at some basal concentration regardless of other species’ metabolism (45, 46). Moreover, decreases in relative concentrations of certain metabolites following antibiotic treatment do not preclude their availability to *C. difficile*. As long as competition for the remaining pool of the given substrate is reduced, *C. difficile* may be able to exploit it as a component of its nutrient niche space. Based on our results, we propose that amino acid catabolism is a primary strategy of *C. difficile in vivo* followed closely by host-derived glycan catabolism. To fulfill its remaining needs, *C. difficile* adapts its metabolism to utilize a combination of carbohydrates, sugar alcohols, or carboxylic acids depending on their availability in the environment. Since the last provide carbon and energy but not nitrogen, it appears that *C. difficile* metabolism strongly prefers nitrogen-containing carbon sources that fulfill a larger proportion of its biological requirements.

Several factors limited our ability to generate transcriptomic replicates for individual mice in each treatment group. Most prominently, we were forced to pool the cecal contents of multiple animals to generate a sufficient quantity of high-quality RNA for extremely deep sequencing that would permit sampling the transcriptome of a rare member of the microbiota (Fig. S2C). Due to possible variation between individual samples that could be masked by this approach, we quantified within-group sample variation for all sample types for which we were able to collect biological replicates. This included *C. difficile* density, 16S rRNA gene abundance, and untargeted mass spectrometry. In order to increase our confidence that transcriptomes were more likely to be consistent between pretreatment groups, we calculated within-group sample variance for *C. difficile* density, 16S rRNA gene abundances, and untargeted metabolomics data sets (Fig. S3B to D). This revealed extremely low variability in each treatment group tested for sample types with increasing levels of complexity. Since these data were collected using matched cecal samples, we were confident that our transcriptomic results reflected reality. Unlike our transcriptomic data, we were able to characterize the metabolome of individual animals; however, these comparisons had their own complications related to the fact that multiple organisms contribute to the overall metabolite pool. The changes observed could be the result of metabolic patterns from other species in each system (host or microbe) in response to pathogen colonization, and it is difficult to discern whether *C. difficile* reaches a density large enough to impact these differences on its own. Possible limitations of our modeling approach also existed, despite much of our results being consistent with previously published work and our own untargeted metabolomic analysis. The metabolite score calculation is dependent on correct and existing gene annotation. In this regard, it has been shown previously that the pathway annotations in KEGG are robust to missing elements (47); however, this does not completely eliminate the possibility of this type of error. Due to the topology of the metabolic network, we were also unable to integrate stoichiometry for each reaction, which may affect rates of consumption or production. Reaction reversibility also varies depending on the versions of enzymes possessed by each species. Since our algorithm favorably weights those metabolites closer to the network periphery, incorrect directionality annotations may lead to mislabeling of reactants or products and potentially lead to incorrect metabolite score calculations. Since our metabolite scoring algorithm selectively amplifies signal for those metabolites with the highest probability of being imported from the environment, this modeling platform may also allow for the identification of emergent properties for the metabolism of *C. difficile* during infection. One example could be the appearance of CO_2_ and formate, apparent metabolic end products, in the list of shared metabolites which scored highly across conditions. Although this may be a shortcoming of the genome or database annotation, one group has posited that *C. difficile* may actually consume CO_2_ under certain conditions and require both of these substrates to undergo this process (48). These findings highlight that our method not only identified growth substrates but also identified additional metabolites that were being utilized for other processes. With further manual curation of the *C. difficile* metabolic network, more species-specific discoveries are possible. Even with this possibility, the application of multiple methods to study the altered physiology of *C. difficile* in mock-infected and infected communities allowed us to validate our results based on known elements of *C. difficile* biology and to internally cross-validate the novel results from our experiments. Ultimately, these results combine to underscore predictions of nutrient niche plasticity.

Our systems approach to studying *C. difficile* metabolism during the infection of susceptible communities combines multiple levels of biological data to identify metabolic trends that would not be apparent by a single method. Only through integrative multiomic analysis of *C. difficile* infection employing genomics, transcriptomics, and metabolomics were we able to uncover a much clearer image of *C. difficile*’s nutrient niche space during infection in the context of complex microbial communities. Focusing on previously established metabolic capabilities of the pathogen, we identify that these forms of metabolism are differentially important to *C. difficile* when colonizing distinct environments. Our data suggest that *C. difficile* is a true bacterial generalist, making it less competitive for specific nutrients against specialists but more fit overall for colonizing a variety of recently vacated nutrient niche spaces. These results have implications for the development of targeted measures to prevent *C. difficile* colonization through pre- or probiotic therapy that will need to be tailored to specific antibiotic-induced perturbations. In the future, this systems-level approach could be expanded to study the niche landscape of entire communities of bacteria and subsequent changes to competition for nutrients in response to antibiotic treatment or pathogen colonization.

## MATERIALS AND METHODS

### Animal care and antibiotic administration.

Six- to 8-week-old GF C57BL/6 mice were obtained from a single breeding colony maintained at the University of Michigan and fed laboratory rodent diet 5001 from LabDiet for all experiments. All animal protocols were approved by the University Committee on Use and Care of Animals at the University of Michigan and carried out in accordance with the approved guidelines. Specified SPF animals were administered one of three antibiotics: cefoperazone, streptomycin, or clindamycin (Table 1). Cefoperazone (0.5 mg/ml) and streptomycin (5.0 mg/ml) were administered in distilled drinking water *ad libitum* for 5 days with a 2-day recovery with untreated distilled drinking water prior to infection. Clindamycin (10 mg/kg of body weight) was given via intraperitoneal injection 24 h before the time of infection. This model was adapted from one previously described (24).

### *C. difficile* infection and necropsy.

All *C. difficile* strain 630 spores were prepared from a single large batch whose concentration was determined a week prior to challenge. On the day of challenge, 1 × 10^3^ *C*. *difficile* spores were administered to mice via oral gavage in phosphate-buffered saline (PBS) vehicle. Subsequent quantitative plating to enumerate the spores was performed to ensure correct dosage. Mock-infected animals were given an oral gavage of 100 μl PBS at the same time as those mice administered *C. difficile* spores. Eighteen hours following infection, mice were euthanized by carbon dioxide asphyxiation and necropsied to obtain the cecal contents. Two 100-μl aliquots were immediately flash-frozen for later DNA extraction and toxin titer analysis, respectively. A third 100-μl aliquot was quickly transferred to an anaerobic chamber for quantification of *C. difficile* abundance. The remaining content in the ceca (approximately 1 ml) was mixed with 1 ml of sterile PBS in a stainless steel mortar housed in a dry ice and ethanol bath. The cecal contents of 9 mice from 3 cages were pooled into the mortar. Pooling cecal contents was necessary so that there would be a sufficient quantity of high-quality rRNA-free RNA for deep sequencing. The pooled content was then finely ground and stored at −80°C for subsequent RNA extraction.

### *C. difficile* cultivation and quantification.

Cecal samples were weighed and serially diluted under anaerobic conditions (6% H, 20% CO_2_, 74% N_2_) with anaerobic PBS. Differential plating was performed to quantify both *C. difficile* spores and vegetative cells by plating diluted samples on CCFAE plates (fructose agar plus cycloserine [0.5%], cefoxitin [0.5%], and erythromycin [0.2%]) at 37°C for 24 h under anaerobic conditions (49). It is important to note that the germination agent taurocholate was omitted from these plates to quantify only vegetative cells. In parallel, undiluted samples were heated at 60°C for 30 min to eliminate vegetative cells and leave only spores (50). These samples were serially diluted under anaerobic conditions in anaerobic PBS and plated on CCFAE with taurocholate (10%) at 37°C for 24 h. Plating was simultaneously done for heated samples on CCFAE to ensure that all vegetative cells had been eliminated.

### *C. difficile* toxin titer assay.

To quantify the titer of toxin in the cecum, a Vero cell rounding assay was performed as described in reference 27. Briefly, filter-sterilized cecal content was serially diluted in PBS and added to Vero cells in a 96-well plate. Plates were blinded and viewed after a 24-h incubation for cell rounding. A more detailed protocol with product information can be found at https://github.com/SchlossLab/Jenior_Modeling_mSystems_2017/blob/master/protocols/toxin_assay/Verocell_ToxinActivity_Assay.Rmd.

### 16S rRNA gene sequencing and read curation.

DNA was extracted from approximately 50 mg of cecal content from each mouse using the PowerSoil-htp 96-well soil DNA isolation kit (Mo Bio Laboratories) and an EpMotion 5075 automated pipetting system (Eppendorf). The V4 region of the bacterial 16S rRNA gene was amplified using custom bar-coded primers and sequenced as described previously using an Illumina MiSeq sequencer (51). All 63 samples were sequenced on a single sequencing run. The 16S rRNA gene sequences were curated using the mothur software package (v1.36), as described previously (51). In short, paired-end reads were merged into contigs, screened for quality, aligned to the SILVA 16S rRNA sequence database, and screened for chimeras. Sequences were classified using a naive Bayesian classifier trained against a 16S rRNA gene training set provided by the Ribosomal Database Project (RDP) (52). Curated sequences were clustered into operational taxonomic units (OTUs) using a 97% similarity cutoff with the average neighbor clustering algorithm. The number of sequences in each sample was rarefied to 2,500 per sample to minimize the effects of uneven sampling.

### RNA extraction, shotgun library preparation, and sequencing.

Pooled, flash-frozen samples were ground with a sterile pestle to a fine powder and scraped into a sterile 50-ml polypropylene conical tube. Samples were stored at −80°C until the time of extraction. Immediately before RNA extraction, 3 ml of lysis buffer (2% SDS, 16 mM EDTA, and 200 mM NaCl) contained in a 50-ml polypropylene conical tube was first heated for 5 min in a boiling water bath (53). The hot lysis buffer was added to the frozen and ground cecal content. The mixture was boiled with periodic vortexing for another 5 min. After boiling, an equal volume of 37°C acid phenol-chloroform was added to the cecal content lysate and incubated at 37°C for 10 min with periodic vortexing. The mixture was then centrifuged at 2,500 × *g* at 4°C for 15 min. The aqueous phase was then transferred to a sterile tube, and an equal volume of acid phenol-chloroform was added. This mixture was vortexed and centrifuged at 2,500 × *g* at 4°C for 5 min. The process was repeated until the aqueous phase was clear. The last extraction was performed with chloroform-isoamyl alcohol to remove the acid phenol. An equal volume of isopropanol was added, and the extracted nucleic acid was incubated overnight at −20°C. The following day, the sample was centrifuged at 12,000 × *g* at 4°C for 45 min. The pellet was washed with 0°C 100% ethanol and resuspended in 200 μl of RNase-free water. Samples were then treated with 2 μl of Turbo DNase for 30 min at 37°C. RNA samples were retrieved using the Zymo Quick-RNA MiniPrep kit. Completion of the DNase reaction was assessed using PCR for the V4 region of the 16S rRNA gene for 30 cycles (51). The quality and integrity of the RNA were measured using the Agilent RNA 6000 Nano kit for total prokaryotic RNA. The Ribo-Zero Gold rRNA removal kit for epidemiology was then used to deplete 16S and 18S rRNA from the samples. Prior to library construction, quality and integrity were measured again using the Agilent RNA 6000 Pico kit. Stranded RNA-Seq libraries were constructed with the TruSeq total RNA library preparation kit, version 2. The Agilent DNA high-sensitivity kit was used to measure concentration and fragment size distribution before sequencing. High-throughput sequencing was performed by the University of Michigan Sequencing Core in Ann Arbor, MI. For all groups, sequencing was repeated across 4 lanes of an Illumina HiSeq 2500 sequencer using 2 by 50-bp chemistry.

### cDNA read curation, mapping, and normalization.

Raw read curation was performed in a two-step process. First, residual 5′ and 3′ Illumina adapter sequences were removed using CutAdapt (54) on a per-library basis. Reads were then quality trimmed using Sickle (N. A. Joshi and J. N. Fass, 2011) on the default settings. An average of ~261,000,000 total reads (both paired and orphaned) remained after quality trimming. Mapping was accomplished using Bowtie2 (55), and the default stringent settings allowing for 0 mismatches again target reference genes. An average of ~6,880,000 reads in each sample mapped to the annotated nucleotide gene sequences of *Clostridioides difficile* 630 from KEGG (Kyoto Encyclopedia of Genes and Genomes) (56). Optical and PCR duplicates were then removed using Picard MarkDuplicates (http://broadinstitute.github.io/picard/), leaving an average of ~167,000 reads per sample for final analysis (see Table S2 in the supplemental material). The remaining mappings were converted to idxstats format using SAMtools (57), and the read counts per gene were tabulated. Discordant pair mappings were discarded, and counts were then normalized to read length and gene length to give a per-base report of gene coverage. Each collection of reads was then subsampled to 90% of the lowest sequence total across the libraries, resulting in even quantities of normalized read abundances in each group to be utilized in downstream analysis. This method was chosen because normalization to housekeeping genes would artificially remove their contributions to metabolic flux and reduce the information provided by our metabolite score calculations within our metabolic modeling approach.

### Reaction annotation and bipartite network construction.

The metabolism of *C. difficile* strain 630 was represented as a directed bipartite graph with both enzymes and metabolites as nodes. Briefly, models were semiautomatically constructed using KEGG (2016 edition) ortholog (KO) gene annotations to which transcripts had been mapped. Reactions that each KEGG ortholog mediates were extracted from ko_reaction.list, located in /kegg/genes/ko/. KOs that do not mediate simple biochemical reactions (e.g., ones that mediate interactions of macromolecules) were omitted. Metabolites linked to each reaction were retrieved from the reaction_mapformula.lst file located in /kegg/ligand/reaction/ from the KEGG release. Those reactions that did not have annotations for the chemical compounds with which they interact were discarded. Metabolites were then associated with each enzyme, and the directionality and reversibility of each biochemical conversion were also saved. This process was repeated for all enzymes in the given bacterial genome, with each enzyme and metabolite node appearing only once. The resulting data structure was an associative array of enzymes associated with lists of both categories of substrates (input and output), which could then be represented as a bipartite network. The final metabolic network of *C. difficile* strain 630 contained a total of 1,205 individual nodes (447 enzymes and 758 substrates) with 2,135 directed edges. Transcriptomic mapping data were then reassociated with the respective enzyme nodes prior to scoring calculations. Betweenness centrality and overall closeness centralization indices were calculated using the igraph R package found at http://igraph.org/r/.

### Metabolite score calculation.

The substrate scoring algorithm (Fig. 3A) favors metabolites that are more likely acquired from the environment (not produced within the network) and awards them a higher score (Fig. 3B and 4A). The presumption of our approach was that enzymes that were more highly transcribed were more likely to utilize the substrates on which they act due to coupled bacterial transcription and translation. The more likely that a compound was to be produced, the more negative the resulting score would be. To calculate the score of a given metabolite (*m*), we used rarefied transcript abundances mapped to respective enzyme nodes. This was represented by *t*_*o*_ and *t*_*i*_ to designate if an enzyme created or utilized *m*, respectively. The first step was to calculate the average expression of enzymes for reactions that either created a given metabolite (i) or consumed that metabolite (ii). For each direction, the sum of transcripts for enzymes connecting to a metabolite was divided by the number of contributing edges (*e*_*o*_ or *e*_*i*_) to normalize for highly connected metabolite nodes. Next, the raw metabolite score was calculated by subtracting the creation value from the consumption value to weight for metabolites that are likely acquired exogenously. The difference was log_2_ transformed for comparability between scores of individual metabolites. This resulted in a final value that reflected the likelihood that a metabolite was acquired from the environment. Untransformed scores that already equaled 0 were ignored, and negative values were accounted for by transformation of the absolute value and then multiplied by −1. These methods have been written into a single Python workflow, along with supporting reference files, and are presented as bigSMALL v1.0 (*b*acter*i*al *g*enome-*s*cale *m*etabolic models for *a*pp*l*ied reverse eco*l*ogy), available in a public GitHub repository at https://github.com/mjenior/bigsmall.

### Transcriptome randomization and probability distribution comparison.

As sequencing replicates of *in vivo* transcriptomes was not feasible, we applied a Monte Carlo-style simulation to distinguish calculated metabolite scores due to distinct transcriptional patterns for the environment measured from those metabolites that were constitutively scored at the extremes of the scale. We employed a 10,000-fold bootstrapping approach of randomly reassigning transcript abundance for enzyme nodes and recalculating metabolite scores. This approach was chosen over fitting a simulated transcriptome to a negative binomial distribution because it created a more relevant standard of comparison for lower coverage sequencing efforts. Using this method, each substrate node accumulated a random probability distribution of metabolite scores, which were then used to calculate the median and confidence interval to generate a probability for each metabolite score to be the result of more than chance. This was a superior approach to switch randomization since the connections of the network itself were created through natural selection, and any large-scale alterations would yield biologically uninformative comparisons (58).

### Anaerobic *in vitro C. difficile* growth curves.

The carbon-free variation of *C. difficile* basal defined medium (NCMM) was prepared as previously described (6). Individual carbohydrate sources were added at a final concentration of 5 mg/ml, and pairwise carbohydrate combinations were added at 2.5 mg/ml each (5 mg/ml total). A solution of the required amino acids was made separately and added when noted at identical concentrations to the same study. Two hundred forty-five microliters of final medium mixes was added to a 96-well sterile clear-bottom plate. A rich medium growth control was also included, consisting of liquid brain heart infusion (BHI) with 0.5% cysteine. All culturing and growth measurements were performed anaerobically in a Coy type B vinyl anaerobic chamber (3.0% H, 5.0% CO_2_, 92.0% N, 0.0% O_2_). *C. difficile* strain 630 was grown for 14 h at 37°C in 3 ml BHI with 0.5% cysteine. Cultures were then centrifuged at 2,000 rpm for 5 min, and resulting pellets were washed twice with sterile, anaerobic phosphate-buffered saline (PBS). Washed pellets were resuspended in 3 ml more PBS, and 5 μl of prepared culture was added to each growth well of the plate containing medium. The plate was then placed in a Tecan Sunrise plate reader. Plates were incubated for 24 h at 37°C with automatic optical density readings at 600 nm (OD_600_) taken every 30 min. OD_600_ values were normalized to readings from wells containing sterile medium of the same type at equal times of incubation. Growth rates and other curve metrics were determined by differentiation analysis of the measured OD_600_ over time in R to obtain the slope at each time point.

### Quantification of *in vivo* metabolite relative concentrations.

Metabolomic analysis was performed by Metabolon (Durham, NC); a brief description of their methods follows. All methods utilized a Waters Acquity ultraperformance liquid chromatograph (UPLC) and a Thermo Scientific Q-Exactive high-resolution/accurate mass spectrometer interfaced with a heated electrospray ionization (HESI-II) source and Orbitrap mass analyzer at 35,000 mass resolution. Samples were dried and then reconstituted in solvents compatible with each of the four methods. The first was under acidic positive conditions using a C_18_ column (Waters UPLC BEH C_18_; 2.1 by 100 mm, 1.7 µm) and using water and methanol, containing 0.05% perfluoropentanoic acid (PFPA) and 0.1% formic acid (FA). The second method was identical to the first but was chromatographically optimized for more hydrophobic compounds. The third approach utilized basic negative-ion-optimized conditions using a separate dedicated C_18_ column. Basic extracts were gradient eluted from the column using methanol and water but, however, with 6.5 mM ammonium bicarbonate at pH 8. Samples were then analyzed via negative ionization following elution from a hydrophilic interaction chromatography column (Waters UPLC BEH amide; 2.1 by 150 mm, 1.7 µm) using a gradient consisting of water and acetonitrile with 10 mM ammonium formate, pH 10.8. The MS analysis alternated between MS and data-dependent MS n scans using dynamic exclusion. The scan range varied slightly between methods but covered 70 to 1,000 *m/z*. Library matches for each compound were checked for each sample and corrected if necessary. Peaks were quantified using area under the curve.

### Statistical methods.

All statistical analyses were performed using R (v.3.2.0). Significant differences between community structures of treatment groups from 16S rRNA gene sequencing were determined with analysis of molecular variance (AMOVA) in the mothur software package. Significant differences of inverse Simpson diversity, CFU, toxin titer, and metabolite concentrations were determined by the Wilcoxon signed-rank test with the Benjamini-Hochberg correction. Undetectable points used half the limit of detection for all statistical calculations. Significant differences for growth curves compared to the no-carbohydrate control (plus amino acids) were calculated using 1-way analysis of variance (ANOVA) with the Benjamini-Hochberg correction.

### Data availability.

Pooled and quality-trimmed transcriptomic read data and experiment metadata are available through the NCBI Sequence Read Archive (SRA) under accession no. PRJNA354635. Data processing steps from the beginning with raw sequence data through the final paper are hosted at http://www.github.com/SchlossLab/Jenior_Modeling_mSystems_2017.
