# Supplementary figures and images for: Clostridium difficile Colonizes Alternative Nutrient Niches during Infection across Distinct Murine Gut Microbiomes
Source: mSystems. 2017 Jul 25;2(4):e00063-17. doi: 10.1128/mSystems.00063-17 (PMC5527303; doi:10.1128/mSystems.00063-17)

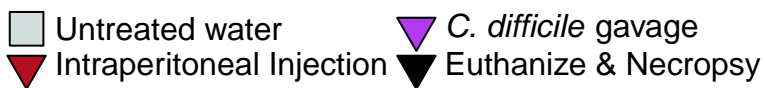

### Streptomycin (SPF)

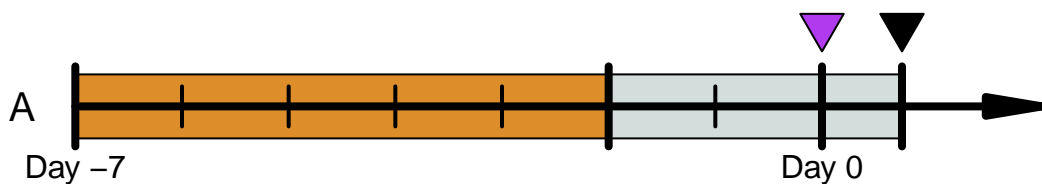

### Cefoperazone (SPF)

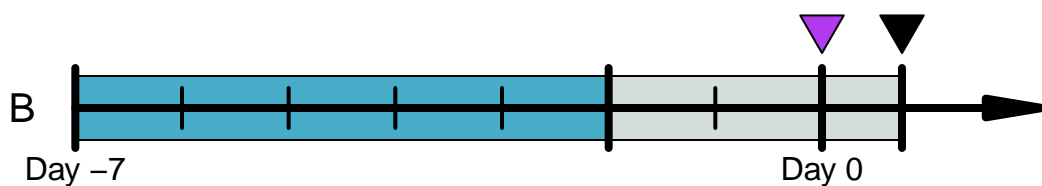

### Clindamycin (SPF)

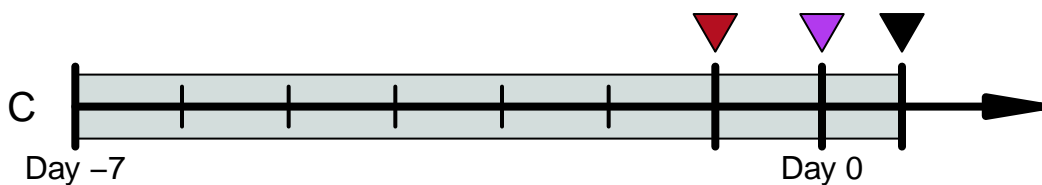

### No Antibiotics (SPF & GF)

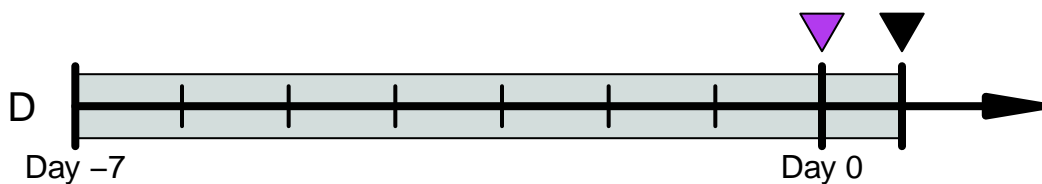

Supplement: FIG S1 [file sys004172122sf1.pdf]

**A**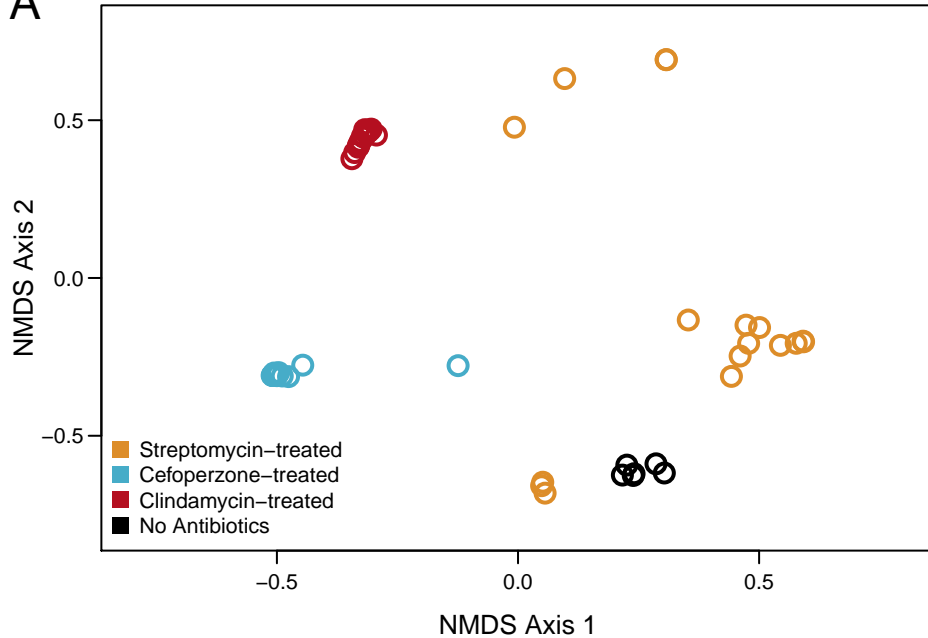**B**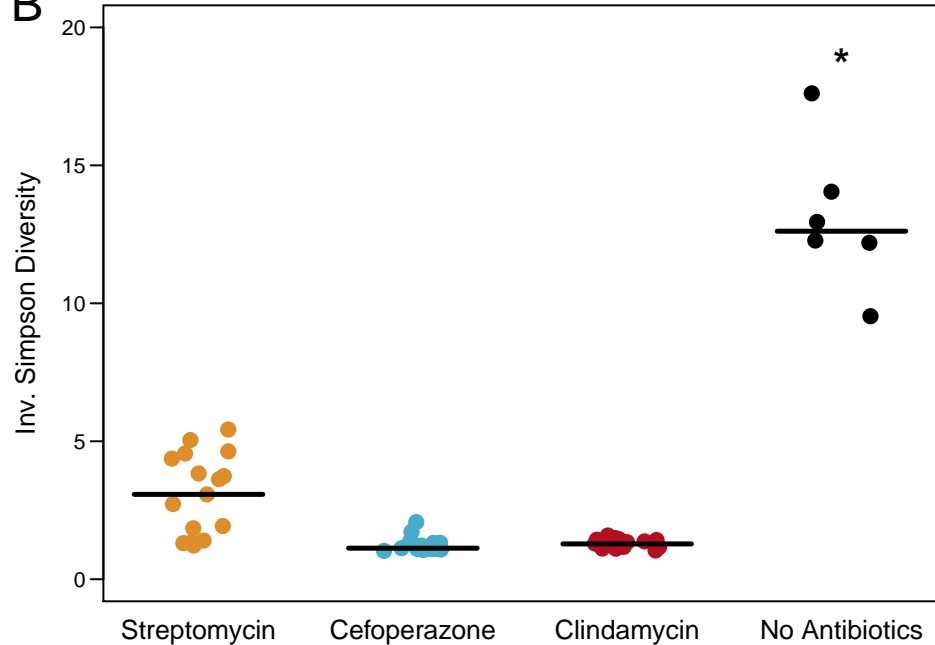**C**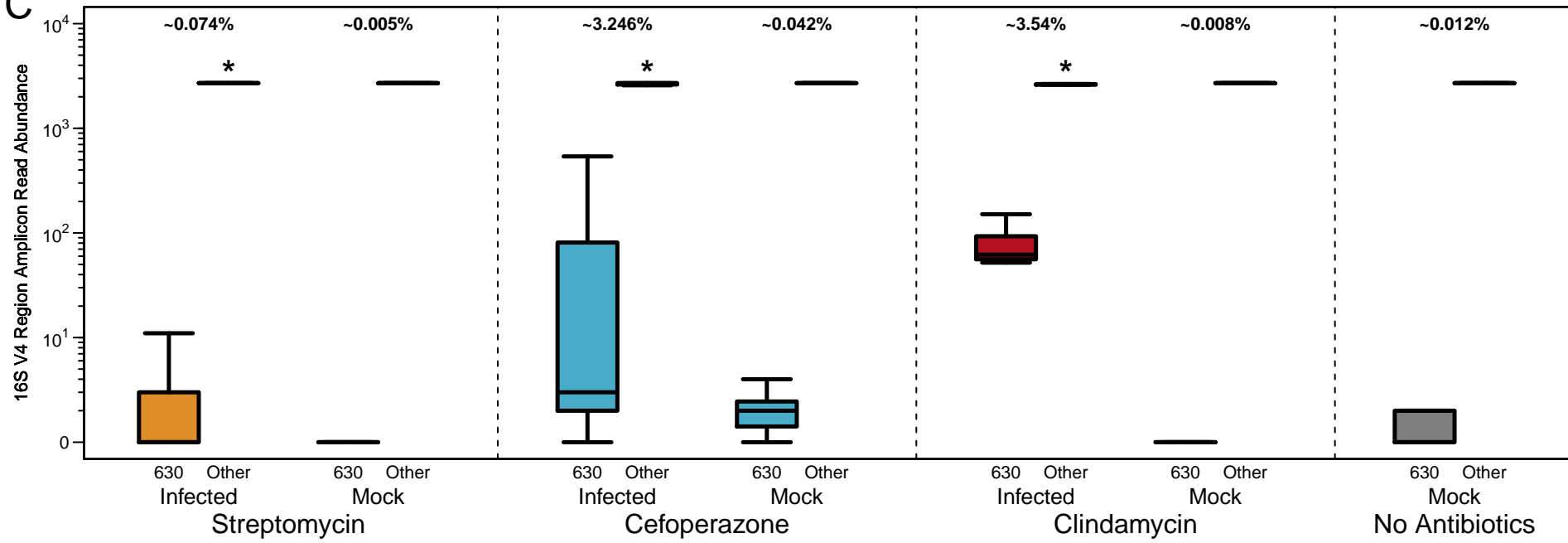

Supplement: FIG S2 [file sys004172122sf2.pdf]

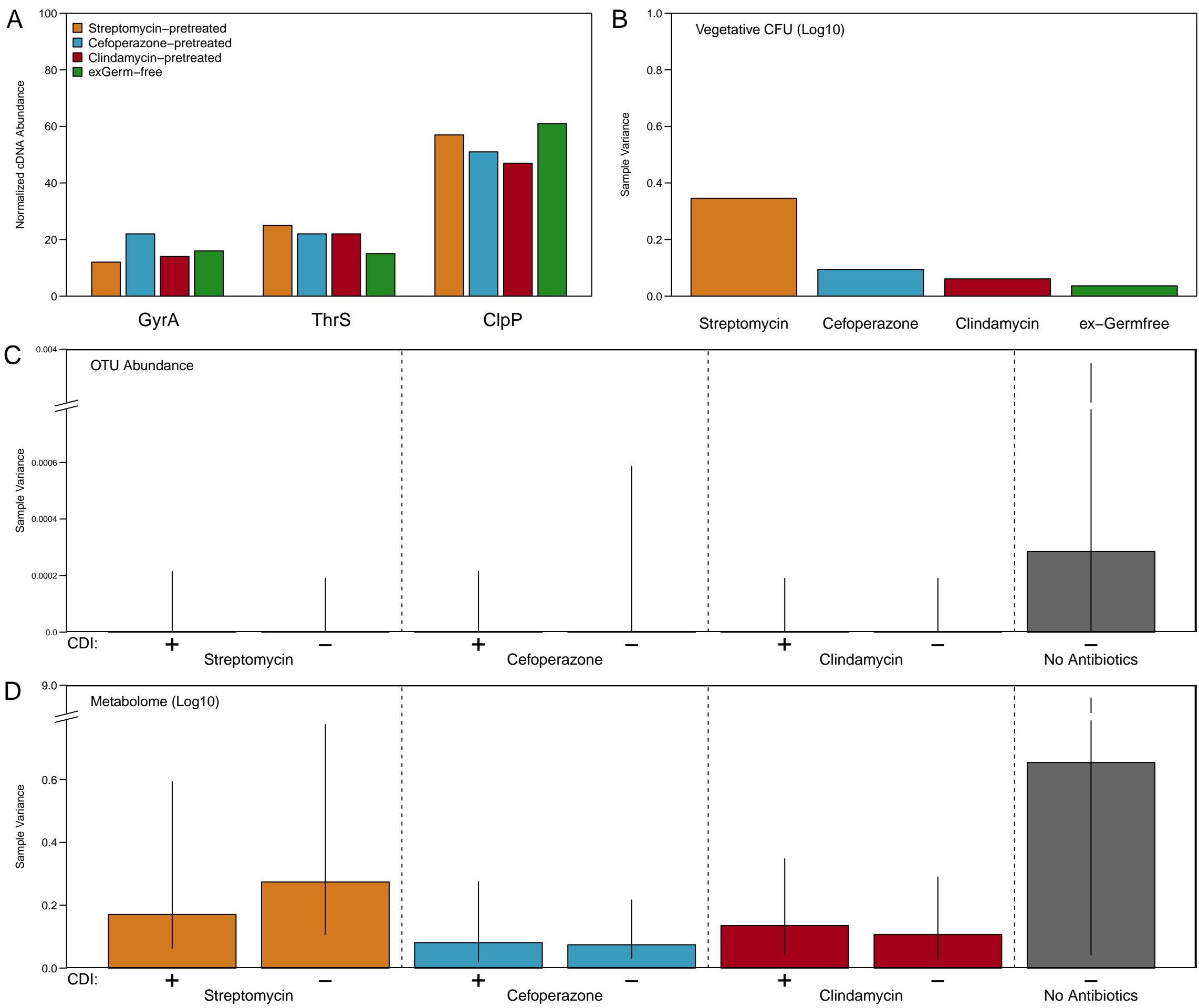

Supplement: FIG S3 [file sys004172122sf3.pdf]

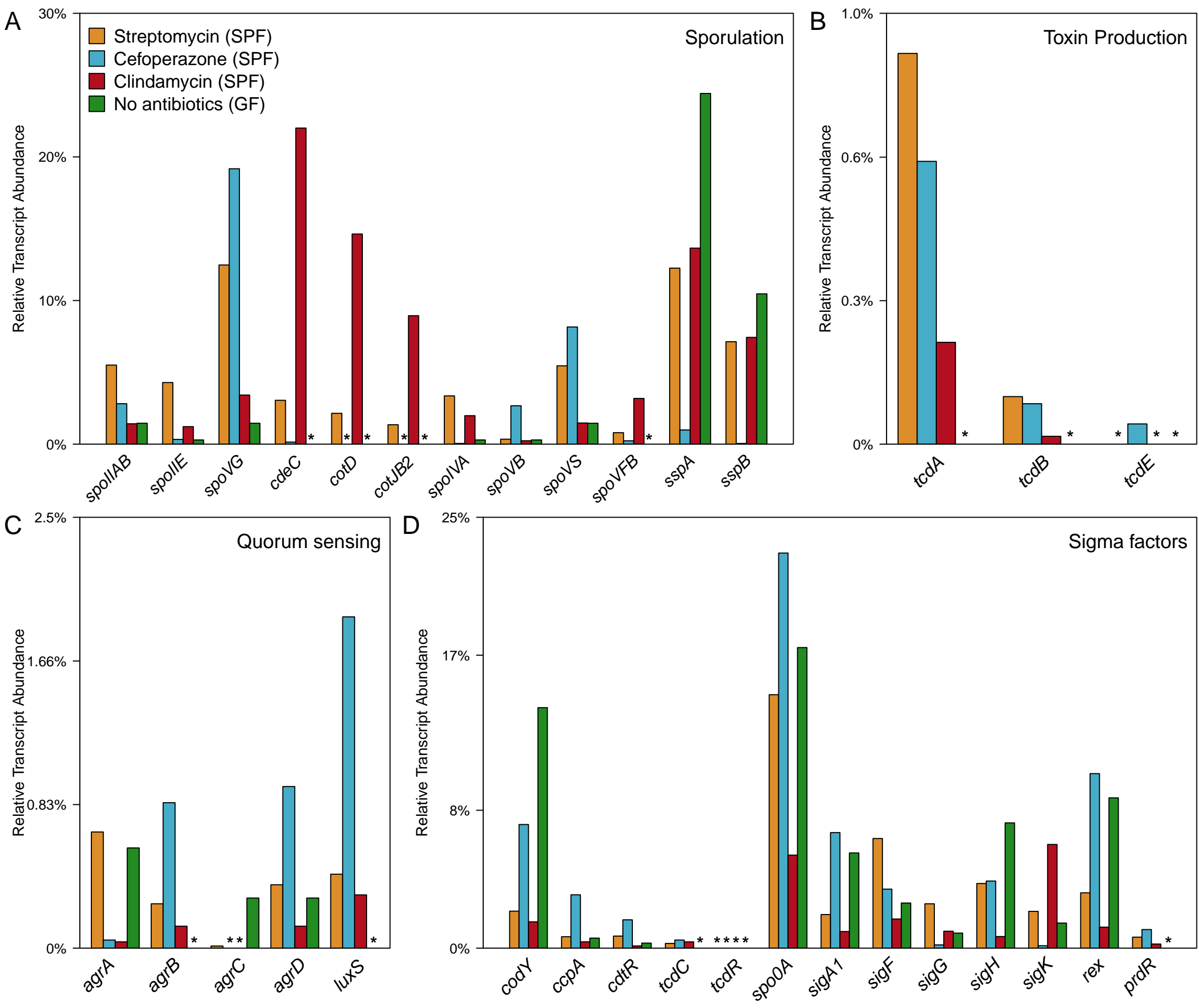

Supplement: FIG S4 [file sys004172122sf4.pdf]

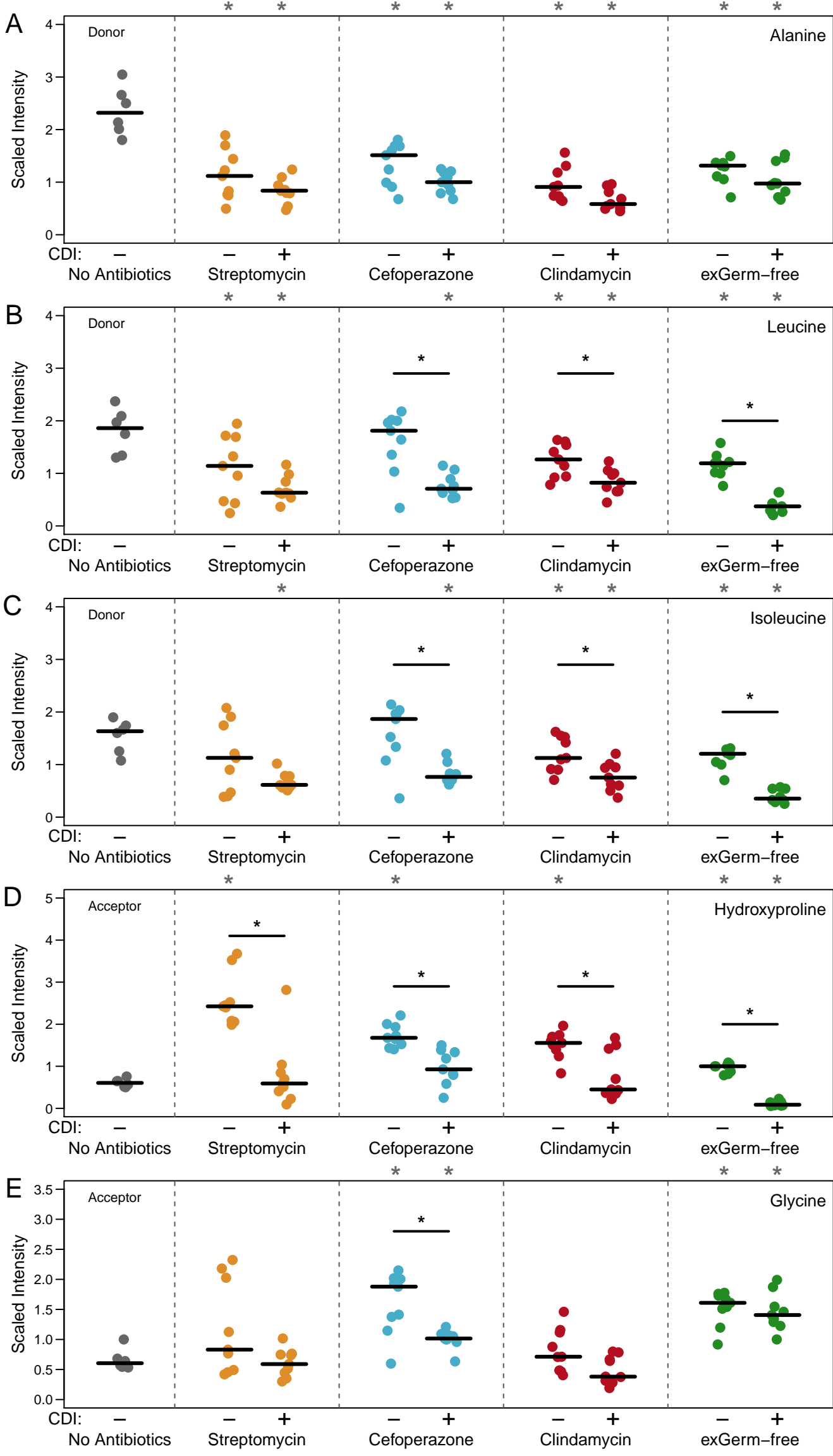

Supplement: FIG S5 [file sys004172122sf5.pdf]
